# In-frame deletion in canine *PITRM1* is associated with a severe early-onset epilepsy, mitochondrial dysfunction and neurodegeneration

**DOI:** 10.1007/s00439-021-02279-y

**Published:** 2021-04-09

**Authors:** Marjo K. Hytönen, Riika Sarviaho, Christopher B. Jackson, Pernilla Syrjä, Tarja Jokinen, Kaspar Matiasek, Marco Rosati, Cristina Dallabona, Enrico Baruffini, Ileana Quintero, Meharji Arumilli, Geoffray Monteuuis, Jonas Donner, Marjukka Anttila, Anu Suomalainen, Laurence A. Bindoff, Hannes Lohi

**Affiliations:** 1 Department of Medical and Clinical Genetics, University of Helsinki, Helsinki, Finland; 2 Folkhälsan Research Center, Helsinki, Finland; 3 Department of Veterinary Biosciences, University of Helsinki, Helsinki, Finland; 4 Department of Biochemistry and Developmental Biology, University of Helsinki, Helsinki, Finland; 5 Department of Equine and Small Animal Medicine, University of Helsinki, Helsinki, Finland; 6 Faculty of Veterinary Medicine, Centre for Clinical Veterinary Medicine, LMU-Munich, Veterinärstrasse 13, 80539 Munich, Germany; 7 Department of Chemistry, Life Sciences and Environmental Sustainability, University of Parma, Parma, Italy; 8 Wisdom Health (Genoscoper Laboratories), Helsinki, Finland; 9 Finnish Food Authority, Helsinki, Finland; 10 Research Programs Unit, Stem Cells and Metabolism Research Program, University of Helsinki, Helsinki, Finland; 11 Department of Clinical Medicine (K1), University of Bergen, Bergen, Norway; 12 Department of Neurology, Neuro-SysMed, Haukeland University Hospital, Bergen, Norway

## Abstract

We investigated the clinical, genetic, and pathological characteristics of a previously unknown severe juvenile brain disorder in several litters of Parson Russel Terriers. The disease started with epileptic seizures at 6–12 weeks of age and progressed rapidly to *status epilepticus* and death or euthanasia. Histopathological changes at autopsy were restricted to the brain. There was severe acute neuronal degeneration and necrosis diffusely affecting the grey matter throughout the brain with extensive intraneuronal mitochondrial crowding and accumulation of amyloid-β (Aβ). Combined homozygosity mapping and genome sequencing revealed an in-frame 6-bp deletion in the nuclear-encoded pitrilysin metallopeptidase 1 (*PITRM1*) encoding for a mitochondrial protease involved in mitochondrial targeting sequence processing and degradation. The 6-bp deletion results in the loss of two amino acid residues in the N-terminal part of PITRM1, potentially affecting protein folding and function. Assessment of the mitochondrial function in the affected brain tissue showed a significant deficiency in respiratory chain function. The functional consequences of the mutation were modeled in yeast and showed impaired growth in permissive conditions and an impaired respiration capacity. Loss-of-function variants in human *PITRM1* result in a childhood-onset progressive amyloidotic neurological syndrome characterized by spinocerebellar ataxia with behavioral, psychiatric and cognitive abnormalities. Homozygous *Pitrm1*-knockout mice are embryonic lethal, while heterozygotes show a progressive, neurodegenerative phenotype characterized by impairment in motor coordination and Aβ deposits. Our study describes a novel early-onset PITRM1-related neurodegenerative canine brain disorder with mitochondrial dysfunction, Aβ accumulation, and lethal epilepsy. The findings highlight the essential role of PITRM1 in neuronal survival and strengthen the connection between mitochondrial dysfunction and neurodegeneration.

**Supplementary Information:**

The online version contains supplementary material available at 10.1007/s00439-021-02279-y.

## Introduction

Mitochondria fulfill multiple metabolic and oxidative phosphorylation-related processes, which are highly tissue- and cell type-specific. Consequently, mitochondrial dysfunction is associated with a broad spectrum of disease presentation including neuropathological susceptibility. The contribution of primary genetic defects or secondarily caused mitochondrial dysfunction in the pathogenesis of neurodegenerative diseases is well recognized in the known examples of Charcot–Marie–Tooth type 2A, Alzheimer’s disease, Parkinson’s disease, and POLG disease (Zuchner et al. 2004; Johri and Beal 2012; Tzoulis et al. 2013).

A recent example of the importance of mitochondrial function in neurons is the report of homozygous missense variants in pitrilysin metallopeptidase 1 (*PITRM1*) causing a neurological syndrome with progressive cerebellar dysfunction and atrophy, with psychiatric manifestations including obsessive behavior, psychosis, and cognitive decline (Alikhani et al. 2011a, b; Brunetti et al. 2016). PITRM1 is a mitochondrial matrix enzyme that processes the targeting sequences of proteins imported across the inner mitochondrial membrane (Falkevall et al. 2006). Failure to degrade these peptides results in their accumulation in the mitochondrial matrix and mitochondrial dysfunction (Alikhani et al. 2011a, b). PITRM1 also appears to degrade short, unstructured peptides and amyloid-β (Aβ) peptides, according to its broad substrate specificity (Falkevall et al. 2006; Brunetti et al. 2016). In mice, a *Pitrm1* knockout is embryonic lethal. However, the heterozygous mice present with a progressive, neurodegenerative phenotype characterized by impaired motor coordination, movement control, and age-dependent accumulation of Aβ deposits in the brain (Brunetti et al. 2016). A more recent human study in brain organoids showed that the loss of PITRM1 function leads to pathology similar to Alzheimer’s disease causing impaired mitochondrial proteostasis and activation of the mitochondrial unfolded protein response (UPR) (Perez et al. 2020). This elicits cytosolic quality control pathways such as the ubiquitin–proteasome system (UPS) and general autophagy. However, with prolonged stress, these mechanisms may not be sufficient to protect neuronal cells against mitochondrial proteotoxicity or overloaded UPS, resulting ultimately in accumulating Aβ deposits and subsequent neurodegeneration. Thus, PITRM1 appears to have a dedicated role in Aβ clearance and even partial impairment of this function can result in neurodegeneration with an accumulation of Aβ, linking the latter with abnormal mitochondrial proteostasis (Brunetti et al. 2016).

Neurodegenerative brain diseases are also common in domestic dogs (*Canis lupus familiaris*) with a high physiological and genetic similarity to the related human conditions (O'Brien and Leeb. 2014). In the present work, we report a novel neurodegenerative disorder in the Parson Russel Terrier breed and describe its clinical and pathological characteristics and the likely genetic cause. The affected dogs develop normally until 6–12 weeks of life before the onset of rapidly worsening seizures, *status epilepticus*, and death. A deletion in *PITRM1* is the probable underlying cause of the disease. Our study sets up a novel canine model of PITRM1-related neurodegenerative disease with a mitochondrial respiratory deficiency and severe epileptic encephalopathy.

## Materials and methods

### Study cohorts

EDTA-blood and tissue samples were collected from privately owned dogs, including 408 Parson Russell Terriers, 88 Fox Terriers, 87 Jack Russell Terriers, 47 Border Terriers and 64 Brazilian Terriers. The study cohort contained seven affected dogs which all had similar seizures with orofacial automatism with facial twitching, repeated jerking head movements, rhythmic blinking, swallowing, salivation, anxious behavior and lethal *status epilepticus*. The samples were stored at − 20 °C until genomic DNA was extracted using the semi-automated Chemagen extraction robot (PerkinElmer Chemagen Technologie GmbH). DNA concentration was determined either with NanoDrop ND-1000 UV/Vis Spectrophotometer or Qubit 3.0 Fluorometer (Thermo Fisher Scientific Inc.). Sample collection was ethically approved by the Animal Ethics Committee of State Provincial Office of Southern Finland (ESAVI/343/04.10.07/2016 and ESAVI/25,696/2020). Pedigrees were compiled by the GenoPro genealogy software and using the public dog registry by the Finnish Kennel Club.

### Clinical and pathological examination

Clinical examinations were performed for one affected puppy and its six littermates at the age of 7.5 weeks. Furthermore, we examined four heterozygous dogs at the mean age of 8.4 years (ranging from 7.3 to 9.4 years). Complete blood count (CBC), serum biochemistry analysis and a complete physical and neurological examination was performed by a board-certified veterinary neurologist for all dogs. In addition, venous blood gases were analyzed in seven puppies (sample taken from *v. jugularis*). A cerebrospinal fluid (CSF) sample was collected by cisternal puncture.

Two dogs (one from Finland, another one from Austria) were subjected to a full postmortem examination. Postmortem examination was performed within 1 h after euthanasia of the male puppy from Finland and within 24 h after euthanasia of the Austrian male puppy. Both brains were harvested after extensive craniectomy, fixed in 10% neutral buffered formalin and trimmed in accordance with IVETF guidelines for epileptic dogs (Berendt et al. 2015). Brain slabs were routinely processed for histology with hematoxylin–eosin (HE), luxol fast blue–cresyl echt violet, Congo red and fluorojade C stainings. Electron microscopy (EM) samples from the cerebral cortex, including several 1 × 1 × 1 mm sized cubes of grey matter from the frontal, parietal, temporal and occipital lobes, were fixed in 2.5% glutaraldehyde and postfixed with 1% osmium tetroxide. Samples were then stained with 8% uranyl acetate in 0.69% maleic acid and embedded in epoxy resin. Ultrathin sections were mounted on copper grids, stained with Reynolds lead citrate, and viewed with a Jeol JEM-1400 (Jeol Ltd., Tokyo, Japan) electron microscope equipped with a Gatan Orius SC 1000B bottom mounted CCD-camera (Gatan Inc., USA) at 80 kV. Immunohistochemical staining (IHC) with primary antibodies directed against Aβ (mouse monoclonal, 17–24 Antibody, Biolegend), PITRM1 (1:500; PAK0154, rabbit polyclonal; LINARIS Biologische Produkte GmbH, Dossenheim, Germany), VDAC1/Porin (rabbit polyclonal, ab15895, Abcam) and Caspase-3 (rabbit polyclonal, LS-C148242, LSBio), glial fibrillary acidic protein (GFAP, mouse monoclonal, MCA1909 Bio-Rad), Ubiquitin (rabbit polyclonal, ab7780, Abcam), Microtubule-associated-light chain 3B (LC3, rabbit polyclonal, ab48394, Abcam), NADH ubiquinone oxidoreductase (Complex I) subunit NDUF A9 (1:50; MS 111, mouse monoclonal; MitoSciences, Eugene, USA); succinate dehydrogenase complex (Complex II) (1:100; MAK6009, mouse monoclonal; LINARIS Biologische Produkte GmbH, Dossenheim, Germany) ubiquinol cytochrome c oxidoreductase (Complex III) subunit Core 2 (1:250; MS 304, mouse monoclonal; MitoSciences, Eugene, USA), cytochrome c oxidoreductase (Complex IV) subunit I (1:500; MS 404, mouse monoclonal; MitoSciences, Eugene, USA), ATP synthase (Complex V) subunit OSCP (1:250; MS 505, mouse monoclonal; MitoSciences, Eugene, USA) was undertaken. For Aβ IHC, sections were pretreated with formic acid for 5 min. Other epitopes were retrieved by incubation in 0.01 M citrate buffer at pH 6 and 99 °C for 20 min. Incubation with primary antibody was at room temperature for 1 h (Aβ) or 2 h (VDAC1/Porin, GFAP, Ubiquitin, LC3) or at 4 °C for 18 h in a humid chamber (respiratory chain complex antibodies). Visualization of positive antibody binding employed either the UltraVision Detection System HRP/DAB kit (Thermo Fisher Scientific Inc.), reacting with mouse and rabbit primary antibodies, using an enzyme-labeled polymer-linked secondary antibody and diaminobenzidine tetrahydrochloride (DAB) as chromogen, or polymers (IMPRESS, LINARIS Biologische Produkte GmbH, Dossenheim, Germany) and a DAB tetrahydrochloride detection kit.

### Homozygosity mapping

Two cases (a male and a female), two unaffected obligate carrier dogs (dam and sire of the affected dogs) and two other unaffected dogs were genotyped using Axiom Canine HD array set A and B containing ~ 1.1 M SNPs (Thermo Fischer Scientific, USA). The genotype data were processed by Axiom Analysis Suite (Thermo Fischer Scientific, USA) and further pruning was performed using PLINK 1.9 (Purcell et al. 2007) with a SNP call rate of > 95%, array call rate of > 95% and minor allele frequency of > 5%. No individual dogs were removed for low genotyping and no SNPs were removed because of significant deviations from the Hardy–Weinberg equilibrium (*p* ≤ 0.0001). After frequency and genotyping pruning, 413,406 SNPs remained for analysis. The homozygosity mapping was performed using PLINK 1.9 with default settings (Purcell et al. 2007).

### Whole-exome and -genome sequencing

The exome libraries for two Parson Russell Terriers, an affected female and an obligate carrier (dam) were prepared with 140702_canFam3_exomeplus_BB_EZ_HX1 kit with a capture size of 152 Mb from the Roche NimbleGen SeqCap EZ target enrichment design (Broeckx et al. 2015). The libraries were sequenced with the Illumina NextSeq500 platform with a read length of 300 bp (paired-end reads, 2 × 150 bp) and coverage of 35X and 40X at the Biomedicum Functional Genomics Unit (University of Helsinki, Finland). The exome sequence data analysis including quality control, mapping, alignment post-processing, single-nucleotide variant calling and small indel calling was performed as described before (Hytönen et al. 2019). An affected Parson Russell Terrier (male) was whole-genome sequenced on Illumina HiSeq X ultra-high-throughput sequencing platform with a read length of 300 bp (paired-end reads, 2 × 150 bp) at the Novogene (HK) Company Limited (Beijing, China). Reads from the WGS sample were processed using SpeedSeq open-source software with bwa (v0.7.17) for alignment to CanFam3.1 assembly, SAMBLASTER for marking duplicate reads, sorting and BAM compression using Sambamba (Chiang et al. 2015).

For WGS samples, variant calling was done using the HaplotypeCaller in gVCF mode, which is combined with gVCFs from our cohort with combineGVCFs and joint genotyping was done by GenotypeGVCFs in Genome Analysis Tool Kit GATK version 4.1 (McKenna et al. 2010). Mobile Element Locator Tool (MELT) was used to detect mobile element insertions (Gardner et al. 2017). The reference sequences of the transposons for MEI discovery were retrieved from the Repbase database (Jurka et al. 2005). DELLY software was used to detect structural variants including deletions, duplications, inversions and insertions by independent commands (Rausch et al. 2012). Functional annotation of variants from both WES and WGS samples was done using Ensembl release-100 and NCBI *Canis lupus familiaris* Annotation Release 105. The identified variants from exomes and WGS were imported into webGQT variant server deployed locally on inhouse canine variant datasets (Arumilli et al. 2020). Subsequently, inheritance model-based candidate variant filtering was performed using webGQT graphical user interface. The variant data were filtered against 645 control genomes (Online Resource 1) assuming an autosomal recessive inheritance of the disease. Prediction of the variant pathogenicity was assessed using PROVEAN (Choi et al. 2012; Choi and Chan 2015) program. The aligned bam files were submitted to SRA with the BioProject accession PRJNA682160. The sample accessions for the two exome samples are SAMN16981797, SAMN16981798 and in addition, SAMN16988768 and SAMN16981799 for the WGS samples. Ensembl transcript ENSCAFT00000008673.4 and UniProt F1PQU2 were used to count the nucleotide and amino acid positions for PITRM1.

### Genomic DNA analysis

Genotyping of individual dogs was performed either by TaqMan assay (Applied Biosystems) or by Sanger sequencing. The following probes flanking the deletion were used for the TaqMan assay: forward primer 5′-CAGGTGACGGCCATTCCT-3′ and reverse primer 5′-CGCCTGTGCGGTCATG-3′. The reactions were run with the Bio-Rad’s CFX96 Touch Real-Time PCR Detection System instrumentation according to the manufacturer’s instructions. For Sanger sequencing a PCR product was amplified using a forward primer (5′-CAGAAAGAGGGTGCGTAGGA-3′) and a reverse primer (5′-CCCCACACCTGAACAAGTTG-3′) flanking the *PITRM1* deletion and AmpliTaq Gold360 Mastermix (Life Technologies). The products were directly sequenced using the PCR primers on an ABI 3730 capillary sequencer (Life Technologies) after treatment with exonuclease I (New England Biolabs) and rapid alkaline phosphatase (Roche). The Sanger sequence data were analyzed using Sequencher 5.4 (GeneCodes).

### RNA expression

Fresh post-mortem samples were collected from the parietal cortex, the cerebellum, the left cardiac ventricular muscle, and the skeletal muscle from one affected Parson Russell Terrier and three age-matched controls in different breeds (Wire Fox Terrier, Saluki and Karelian Bear Dog). All dogs were euthanized for humane reasons with owners’ consent. The affected Parson Russell Terrier was euthanized at the age of 2 months and 7 days due to severe seizures and *status epilepticus*; a female Wire Fox Terrier was euthanized at the age of 1 month and 25 days due to van den Ende-Gupta syndrome; a male Saluki puppy at the age of 2 months and 5 days due to malocclusion, and a male Karelian Bear Dog at the age of 2 months and 22 days due to severe skeletal abnormalities. The tissue samples were harvested immediately after euthanasia and snap-frozen in liquid nitrogen before storing at − 80 °C. Total RNA was extracted using the RNeasy Mini Kit (Qiagen). Sample concentrations were measured using DeNovix DS-11 Spectrophotometer (DeNovix Inc. Wilmington, Delaware, USA). The high-capacity RNA-to-cDNA Kit (Applied Biosystems, Life Technologies) was then used to reverse-transcribe equal amounts of total RNA into cDNA. The canine PITRM1 transcript was amplified and sequenced using a primer pair (GGTTAAGATGTGCGCTCAAG and CGCGATCCTCTTCATCAACT) that was designed to span an exon–intron boundary to avoid genomic DNA contamination. Quantitative PCR was performed in triplicates for each sample using Bio-Rad’s CFX384 Touch Real-Time PCR Detection System instrumentation according to the manufacturer’s instructions. Tyrosine 3-Monooxygenase/Tryptophan 5-Monooxygenase Activation Protein Zeta (YWHAZ) and GAPDH were used as loading controls. The relative normalized expression levels were calculated with Bio-Rad’s CFX Manager Software. Outliers were removed, except for the cerebellum sample from the Wire Fox Terrier, in which the standard error between the three samples was as high as 0.29, but no clear outlier was seen. Standard error was under 0.2 for all other samples. In further analysis, a mean of the three controls was used when assessing relative expression level to case. A *t* test was performed to compare expression levels in the controls (*n* = 3) and case (*n* = 1) in the four tested tissues. *P *values were calculated expecting a similar expression pattern between the case and controls and using a two-sample *t* test calculator by Usable Statistics (https://www.usablestats.com/calcs/2samplet&summary=1).

### Cell culturing and immortalization

Fibroblasts from one affected dog and one age-matched control (a female Wire Fox Terrier) were cultured in high glucose DMEM medium with 4.5 g/l glucose (Lonza, Cat. No. 12-614Q) having 10% fetal bovine serum (Gibco Life Technology, Ref. 10500–064), 2 mM GlutaMAX (Gibco Life Technology, Ref.35050–38), and 100 U/ml penicillin–streptomycin (Gibco Life Technology, Ref. 15140-122). The cells were immortalized using a retrovirus packaging cell line PA317 (host range: amphitropic), transfected with a construct expressing E6 and E7 of HPV16. The retroviral immortalization was induced at 30–50% confluency in 10 cm diameter plates by removing media and adding 1 ml of virus stock and 3 ml of media with 4 µg/ml polybrene. The plates were incubated at 37 °C for 4 h, after which additional 5 ml of polybrene-containing media was added. Incubation was continued for 2 more hours. The media was removed, and the cells were supplemented with polybrene-free media after rinsing. At day 3, cells were supplemented with 50 µg/ml G418 to select transduced cells and continued for 10 days.

### Protein extraction

Normal skin fibroblasts were collected by trypsinization and washed with 1 × PBS and pelleted at 2000 × g for 5 min. The cells were then lysed in Extraction Reagent T-Per (Thermo Fisher Scientific) containing protease inhibitor cocktail (Thermo Fisher Scientific). The lysis was carried out by 3 cycles of freezing and thawing and homogenized by passing the solution 10 times through a 21 Gauge needle. The homogenate was centrifuged 15 min at 4 ºC and 12,000×*g*. The protein concentration was determined with the BCA kit (Thermo Fisher Scientific) following the manufacturer instructions. All homogenates were stored at – 80 ºC until further use. Brain cortex, muscle, liver, and cerebellum were obtained during necropsy, snap-frozen in liquid nitrogen, and stored at – 80 ºC. Tissues were homogenized in Extraction Reagent T-Per containing protease inhibitor cocktail by disruption for 30 s with TissueRuptor (QIAGEN). The tissue homogenates were processed identically to the cell homogenates, protein concentration determined and stored at – 80 ºC.

### Immunoblotting

Equal amounts of proteins were run on 4–20% Mini-PROTEAN TGX Stain-Free Protein Gels (Bio-Rad) and blotted onto nitrocellulose membrane using a Mini Trans-Blot Cell system (Bio-Rad). Membranes were blocked for 1 h at room temperature (RT) with a filtered solution of 4% skimmed milk in 1xTBS containing 1% Tween20. After blocking, membranes were rinsed with TBS-T and incubated overnight at 4 ºC with primary antibodies (α-mt-Co1 Abcam #14705, α-Tom20 Abcam #186735, α-Vinculin Abcam #129002, α-ATP5A Abcam #14748, Sdha Abcam #14715, α-Pitrm1 Linaris #PAK0154). After washing in TBS-T, secondary HRP-coupled antibodies were incubated for 1 h at RT (1:5000 goat anti-rabbit and 1:2000 goat anti-mouse antibodies). Signal detection was performed with Clarity Western ECL Substrate (Bio-Rad) following the manufacturer’s instructions on a Gel Doc XR + Gel Documentation System (Bio-Rad).

### High-resolution respirometry

For tissue samples, the brain of euthanized dogs was immediately removed, and appropriate frontal cortex of cerebellum tissue pieces placed on ice in respiration buffer (110 mM d-sucrose, 60 mM lactobionic acid, 20 mM HEPES, 20 mM taurine, 10 mM KH_2_PO_4_, 3 mM MgCl_2_, 0.5 mM EGTA, 1 g/l fatty acid-free BSA, pH 7.1). 20–25 mg (wet weight) of each tissue was lysed in respiration buffer with 7–8 × strokes using a handheld Teflon Potter–Elvehjem. Sampling times were kept constant, and all samples were measured in duplicates. A total of 2 mg per sample was measured in a substrate-uncoupler inhibitor protocol using an Oroboros high-resolution oxygraph. Oxygen consumption rates were assessed in the presence of pyruvate-glutamate-malate with specific activities determined with + ADP, and + succinate (CI&CII-linked maximal coupled respiration, state III), maximal uncoupled respiration by titration of FCCP and individual assessment of complex IV by + Asc/TMPD (CIV). Rates of oxygen consumption are expressed as pmol/s normalized to wet weight of tissue or cell amount as described before (Jackson et al. 2014).

### Yeast studies

Modeling in yeast was performed using strain W303-1B *cym1Δ* (*MATα ade2-1 ura3-1 his3-11 trp1-1 leu2-3,112 can1-100 cym1::KANMX4*) (Brunetti et al. 2016)*.* All experiments, except transformation, were performed in synthetic complete (SC) media (0.69% YNB without amino acids (ForMedium, UK), 1 g/l dropout mix without amino acids or bases necessary to keep plasmids). Media were supplemented with various carbon sources (Carlo Erba Reagents, Italy) as indicated in the results and figures in liquid phase or after solidification with 20 g/l agar (ForMedium, UK). Growth was performed with constant shaking at 37 °C.

Mutations were introduced in pFL38CYM1HA plasmid (Brunetti et al. 2016) trough Quikchange PCR using the primers CYM1R41SFw (5´-gaattctaccggttccggagTtgagCctcactgcggtagatttgg-3) and CYM1R41SRv (5´-ccaaatctaccgcagtgagGctcaActccggaaccggtagaattc-3) or CYM1L40R41delFw (5`-gaagaattctaccggttccggagctcactgcggtagatttggtg-3) and CYM1L40R41delRv (5´-caccaaatctaccgcagtgagctccggaaccggtagaattcttc-3). Plasmids harboring *CYM1* mutations were introduced in the W303-1B *cym1Δ* strain through LiAc/ssDNA/PEG transformation method (Gietz 2014). Spot assays, oxygen consumption rate and western blot were performed as previously reported (Brunetti et al. 2016), except that in western blot a rat anti-HA (1/2000) and a mouse anti-Por1 (1/10,000) antibody (Abcam, UK) were used as primary antibodies and Goat anti-Rat IgG DyLight 650 (Thermo Fisher Scientific Inc, USA, 1/4000) and Goat Anti-Mouse IgG SB Blue520 (Bio-Rad Laboratories, USA, 1/10,000) were used as secondary antibodies. Signal was detected using the Chemidoc MP Imaging System (Bio-Rad Laboratories, US) and quantified with Image Lab software.

## Results

### Clinicopathological analyses find lethal epilepsy and neurodegeneration

**Fig. 1:**
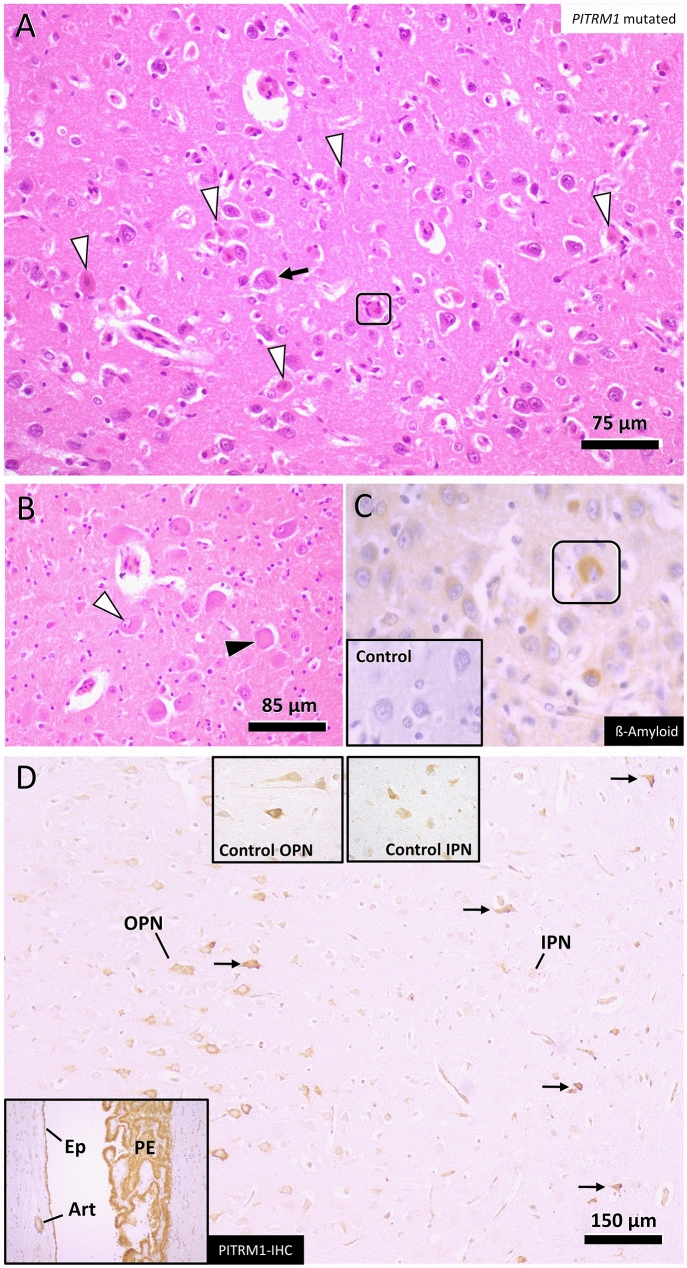
Representative forebrain changes in a PITRM1 mutant dog. **a** Extensive eosinophilic nerve cell necroses are seen within the cerebral cortex (arrowheads) some of which have triggered early microglial neuronophagia (frame). Surrounding non-necrotic changes also show degenerative cytoplasmic and nuclear features such as lobulated giant nuclei (black arrow). **b** Multiple diencephalic neurons further show central chromatolysis (black arrowhead) and nuclear displacement (white arrowhead). **c** Immunohistochemistry for Aβ stains some neurons within the necrotic cortical areas (frame). No such staining was identified in age-matched controls (inlet). **d** Throughout the cortices groups of outer (OPN) and inner pyramidal neurons (IPN) stain immunopositive for PITRM1 antigen similar to control dog sections (upper inlets). This also applies to neurons with degenerate features (arrows). Choroid plexus epithelium (PE), ependymal (Ep) and arterial smooth muscle layers (Art) serve as internal positive control due to constitutive expression of PITRM1 (lower inlet)

A litter of five puppies was born to healthy Parson Russell Terrier parents in Finland. All of them were normal until 7 weeks of age when two of the puppies started having seizures. A typical seizure included orofacial automatism with facial twitching, repeated jerking head movements, rhythmic blinking, swallowing, salivation and anxious behavior (Online Resource 2 and 3). Consciousness was decreased during the episodes, which progressed to *status epilepticus* and death of one affected puppy. The other affected littermate had a similar clinical picture, and the dog was euthanized soon after. Later, we learned of four other litters from Austria and Finland with altogether ten affected puppies (five females and five males) having above-mentioned clinical features, which started between 6 and 11 weeks of age. All of them died or were euthanized within a few days after the onset due to *status epilepticus*. Postmortem examination of one affected Austrian puppy revealed an acute diffuse forebrain-predominant necrotizing polioencephalopathy affecting mainly the pyramidal cell layers of hippocampus proper and entorhinal cortex, followed by those of frontal, parietal, temporal, and occipital neocortex as well as of thalamic nuclei (Fig. 1a). Neuronal necroses were of Fluorojade C positive excitotoxic type and accompanied by spatially variable stages and degrees of astrogliosis, microgliosis and capillary prominence. Next to necrotic neurons, many nerve cells underwent central chromatolysis with nuclear displacement (Fig. 1b). Moreover, a diffuse partially cell-bound (astrocytic) reabsorption oedema was seen throughout the affected cortices and nuclei. Sparsely, there was evidence of individual Purkinje cell necrosis in the cerebellum.

### Genetic analyses reveal an in-frame deletion in *PITRM1*

**Fig. 2:**
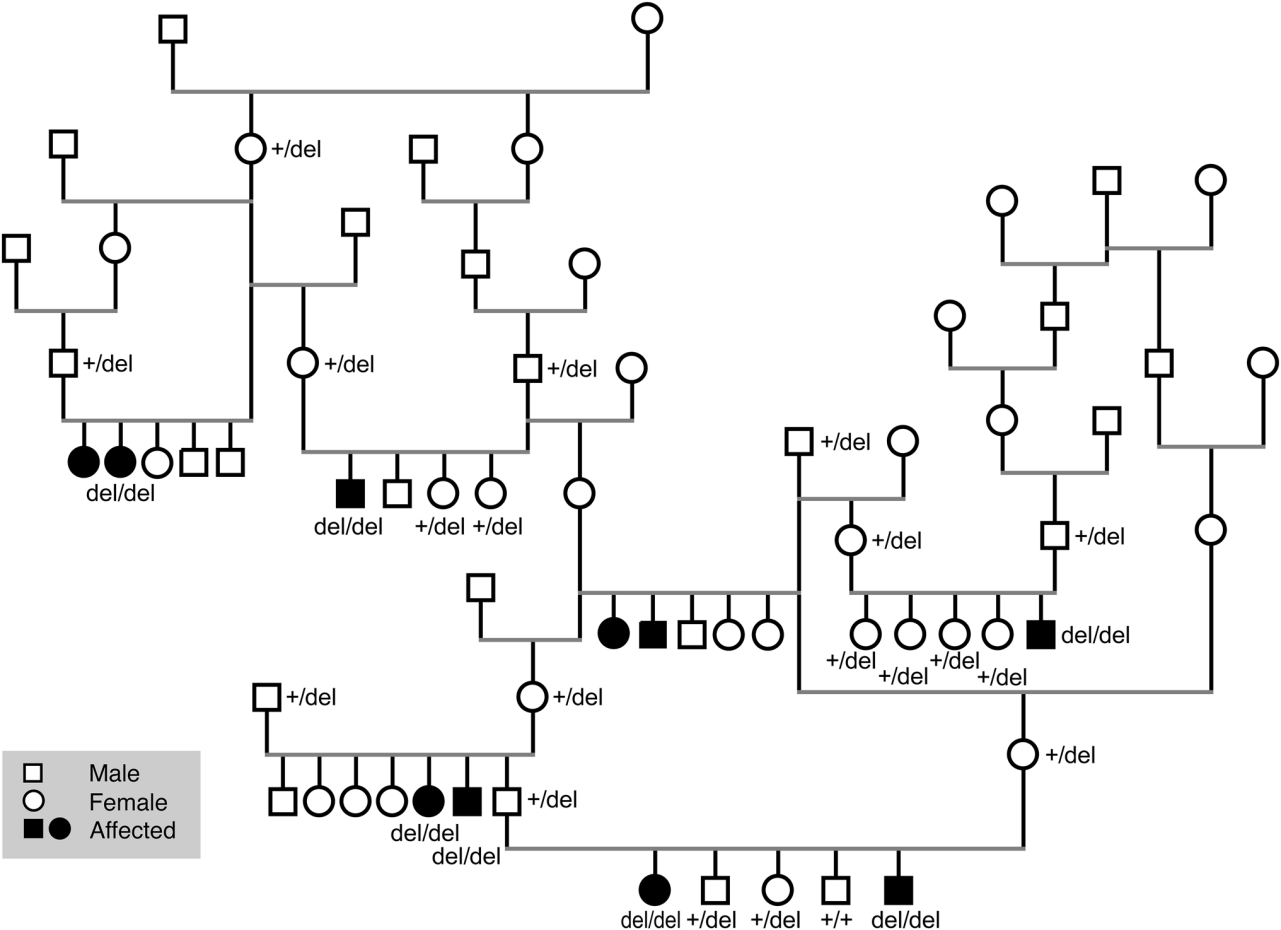
A pedigree established around the affected dogs. The affected dogs are closely related. Genotype information of the PITRM1 allele is denoted for each genotyped dog. The variant segregates in the pedigree as expected in a recessive disease

**Fig. 3:**
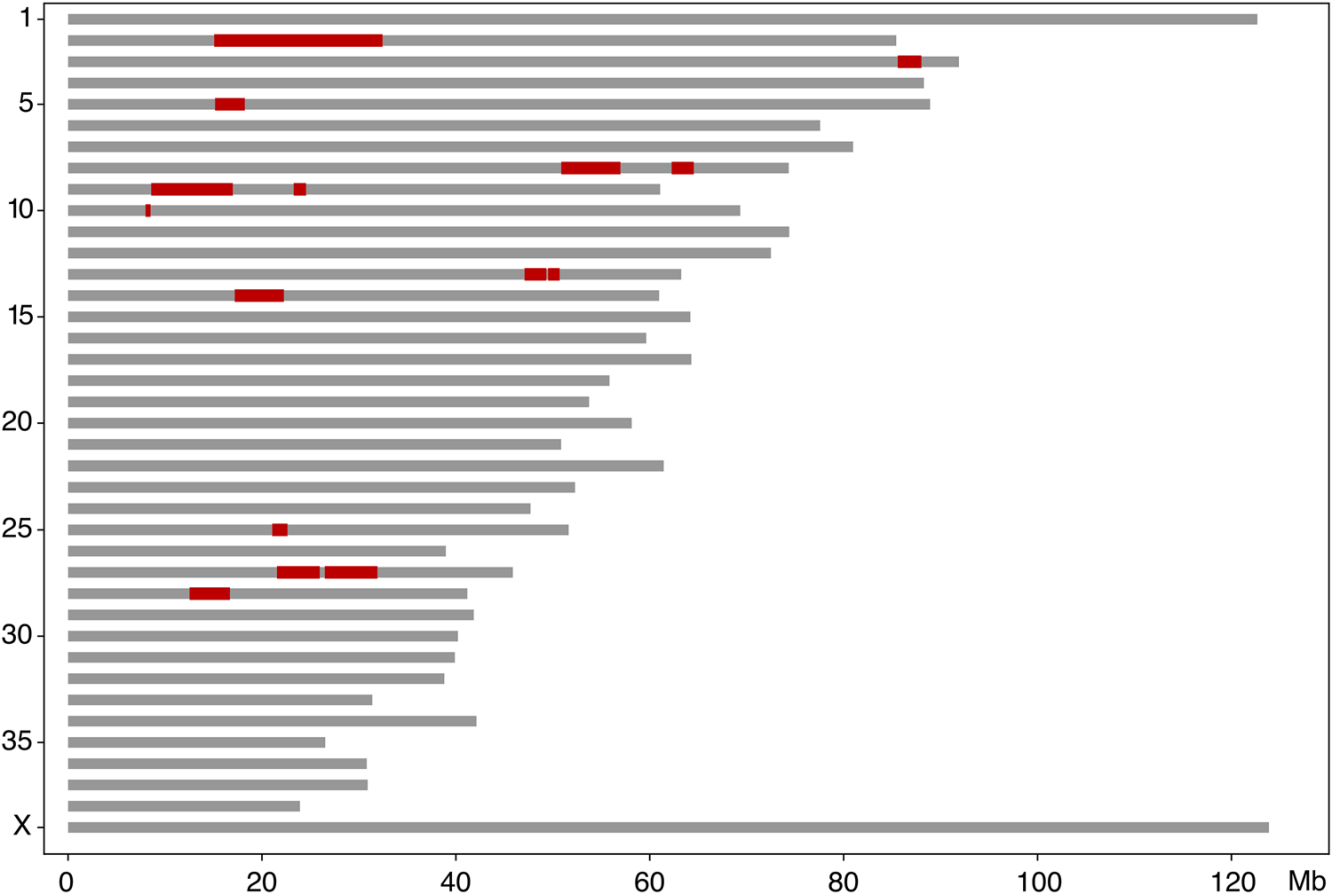
Homozygosity mapping of two affected and four unaffected dogs. The analysis resulted in a total of 15 case-specific regions of homozygosity (indicated in red)

Relatedness information of the affected dogs suggested an autosomal recessive mode of inheritance for the disease as all affected dogs were closely related, including affected littermates, both sexes were affected, and parents were unaffected (Fig. 2). We carried out genome wide analysis to map the candidate loci and genotyped two affected and four control dogs using Axiom canine genotyping arrays with ~ 1.1 M SNPs. Homozygosity mapping resulted in 15 runs of homozygosity (ROH) specific for the cases (Fig. 3 and Online Resource 4), the largest region being 17 Mb on chromosome 2 spanning from 15,171,026 to 32,339,971 bp.

Subsequently, we performed whole-exome sequencing (WES) on one affected dog and one unaffected obligate carrier dog (parent). We compared the variants against variant data from 545 unaffected dogs from different breeds according to a recessive inheritance model. Only 19 variants remained after filtering and only one of them resided in the ROH gained from homozygosity mapping (Online Resource 5). The variant is an in-frame 6-bp deletion in pitrilysin metallopeptidase 1 (ENSCAFT00000008673 c.175_180del), a known neurodegenerative disease gene (Brunetti et al. 2016). We also carried out whole-genome sequencing (WGS) on another case to confirm the *PITRM1* variant and exclude possible structural variants and mobile element insertions within the ROH. Filtering of the variant data (SNVs and indels) of the affected dog against 545 unaffected dogs resulted in a total of 3045 homozygous variants from which 38 were predicted to affect protein (Ensembl annotated genes) (Online Resource 6). Again, only the 6-bp deletion in *PITRM1* resided within the ROH loci. We carried out the filtering of mobile element insertions and structural variants against 290 control genomes and found 47 case-specific variants in total (Online Resource 7). Two of the structural variants resided within mapped ROH loci but outside coding regions and were, therefore, excluded.

**Fig. 4:**
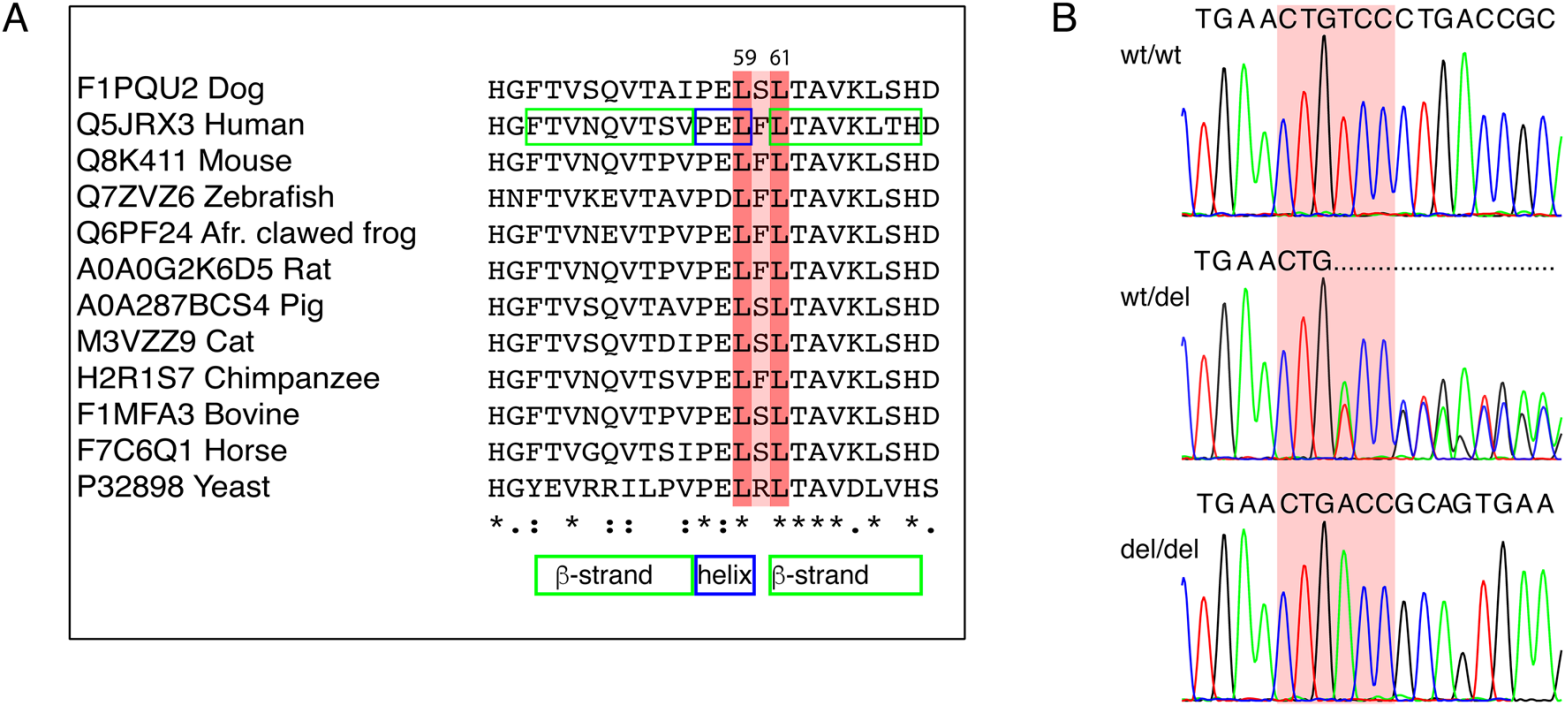
**a** Multiple alignment of PITRM1 orthologs spanning the predicted deletion site, p.(Leu59_Ser60del) or alternatively p.(Ser60_Leu61del) and surrounding amino acids demonstrates the conservation of the Leu59 as well as alternatively deleted Leu61 across species (Leu highlighted in red and Ser in light red). In human, the corresponding amino acids overlap a junction between a short helix (blue rectangle) and β-strand (green rectangle). **b** Sequence chromatograms illustrating the *PITRM1* deletion allele as homozygous (del/del) in an affected dog, heterozygous (wt/del) in an unaffected obligate carrier dog and homozygous wild-type (wt/wt) allele in an unaffected dog. Deletion site (reference allele CTGTCC) is highlighted in red

The 6-bp deletion in *PITRM1* results in the loss of two amino acid residues in the N-terminal part of the protein and can be predicted as p.(Leu59_Ser60del) or alternatively p.(Ser60_Leu61del). The deletion site overlaps a region where the predicted three-dimensional structure forms a short helix followed by a β-strand. In silico analysis of the variant using PROVEAN Protein tool predicted the variant to be a deleterious change with a score of − 12.130 (cutoff − 2.5). Multiple alignment of the protein orthologues demonstrates that the predicted residues of the deletion, Leu59 as well as Leu61, are highly conserved across species, including yeast (Fig. 4).

We then genotyped 410 Parson Russell Terriers, including all 7 affected and 401 unaffected dogs, to study the variant’s segregation and breed-specificity (Fig. 4). We found 332 wild-type, 69 heterozygous and 7 homozygous dogs while all the affected dogs’ parents (*n* = 11) were carriers (Fig. 2). These results show full segregation of the variant according to a recessive model with the carrier frequency of 17% in the cohort. Additional genotyping of 286 dogs from 4 related Terrier breeds showed them all as homozygous for the wild-type allele. We also had access to variant frequencies from commercially tested dogs, including 28,169 dogs from 374 breeds or breed variants (Online Resource 8). Only 21 heterozygous dogs were found, and they were all Parson Russel Terriers which resulted in a carrier frequency of 5.2% in the breed cohort.

### The *PITRM1* deletion transcript and protein are expressed

**Fig. 5:**
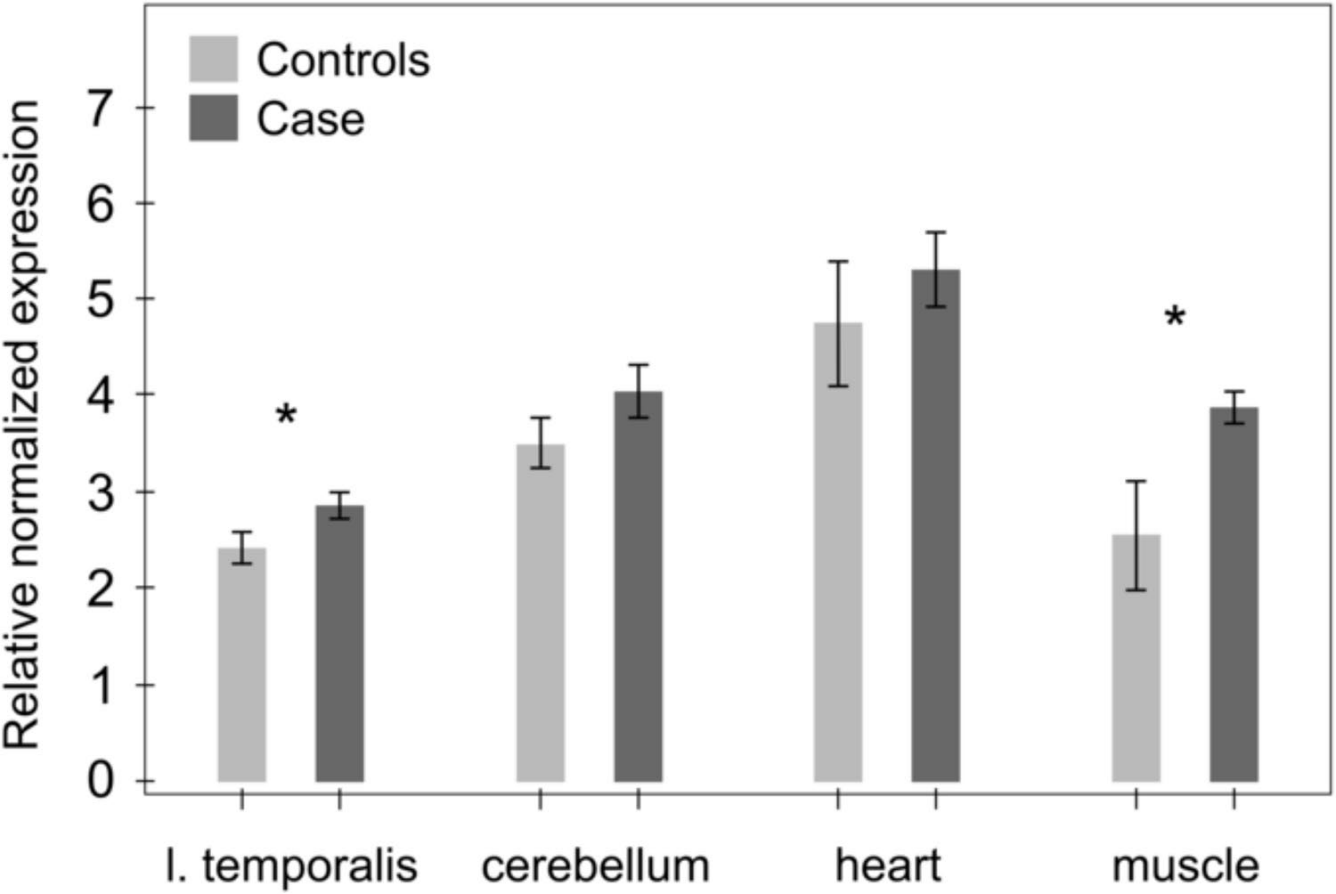
Comparison of the *PITRM1* transcript levels between a case and control dogs. The stability of the *PITRM1* transcript was studied by a quantitative PCR in four different tissues (parietal cortex, cerebellum, cardiac muscle and skeletal muscle) in one (~ 2 months old) affected and three unaffected dogs (Control 1 = Wire Fox Terrier; Control 2 = Saluki; Control 3 = Karelian Bear Hound). The results suggest increased expression of the aberrant transcript in the parietal cortex and the skeletal muscle tissues. The error bars refer to variance in experimental tripli- or duplicates and between the three control dogs. The asterisks refer to a *P *value under 0.05 in a two-sample *t* test. YWHAZ and GADPH were used for normalization

We next wanted to test whether the 6-bp deletion affected the stability of the *PITRM1* transcript. The expression was measured in the RNA samples isolated from the parietal cortex, the cerebellum, the left cardiac ventricle, and the skeletal muscle from one affected and three unaffected dogs. *P* values calculated with a two-sample *t* test between the controls and case for the parietal cortex, the cerebellum, the left cardiac ventricle, and the skeletal muscle tissues were 0.02, 0.09, 0.33 and 0.04, respectively. These results suggest a slightly increased expression of the mutant transcript in the parietal cortex and the skeletal muscle tissues (Fig. 5), but our overall sample size is too small for definitive conclusions.

PITRM1 expression in the brain was assessed by IHC. The staining was comparable between affected animals and control dogs. Plexus epithelia, ependymal cells and arterial smooth muscle cells (mediacytes) stained strongly throughout all sections (Fig. 1d). Neuronal expression depended on both type of neurons as well as on their topographic localization. The most intense staining was observed in large multipolar neurons of brain stem, pyramidal cells of cerebral cortices and Golgi cells of cerebellar granular layer. Therein, immunopositivity frequently highlighted the Golgi zone or stained the entire perikaryon in a granular fashion. PITRM1 expression was noted in both necrotizing neurons as well as in spared ones (Fig. 1d).

### Clinical follow-up and histopathology of a puppy with a homozygous *PITRM1* deletion

During the study, we identified a newborn litter with one homozygous male and four heterozygous females. We examined the entire litter for the first time at the age of 7.5 weeks. According to the breeder, all puppies were normal at this stage. The physical and neurological examination showed no abnormalities and blood cell count (CBC), serum biochemistry, venous blood gases, and lactate levels were normal.

At the age of 10 weeks, the homozygous puppy developed a cluster of seizures. The puppy continued to have focal-onset seizures despite diazepam and loading dose of phenobarbital (Online Resource 9) and was sedated with dexmedetomidine. After recovering from the sedation, the puppy started to have seizures again. Cell count and protein content of the cerebrospinal fluid (CSF) sample were normal. The puppy was euthanized due to pharmacoresistant seizures.

**Fig. 6:**
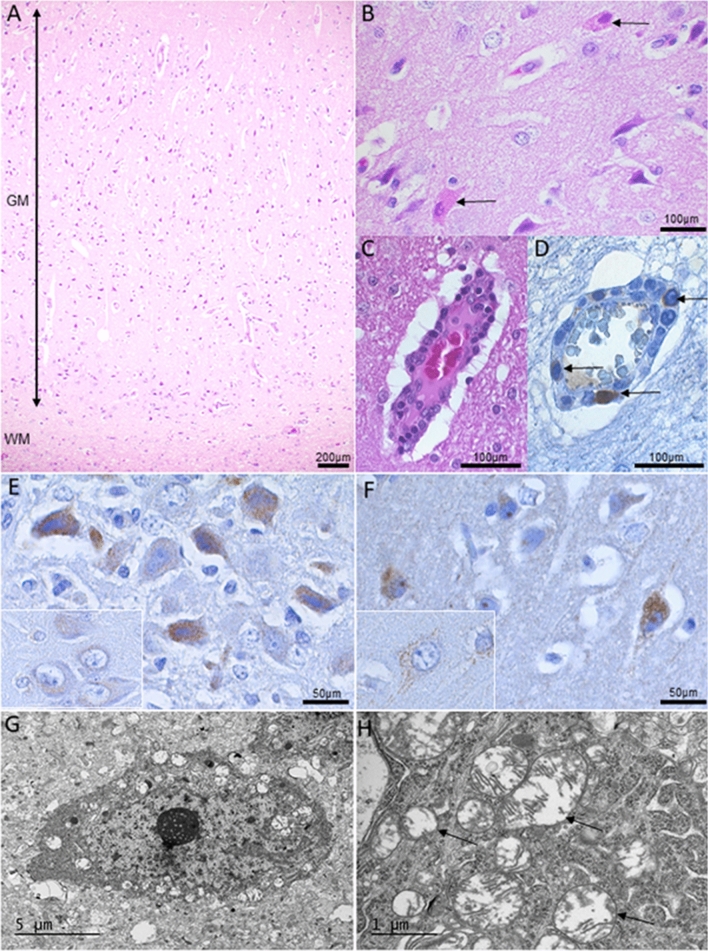
Histopathological findings in a dog affected by PITRM-associated mitochondrial juvenile encephalopathy. **a** Widespread acute degeneration and necrosis of cerebral grey matter (GM). **a** HE, WM: white matter. **b** Shrunken, granular and eosinophilic degenerating neurons (arrows) of the cerebral cortex. HE. **c** Early inflammatory response to the neuronal necrosis seen as increased cellularity of cortical vessel walls. HE. (**d**) Vascular wall degeneration with scattered intramural apoptotic cells (arrows). IHC Caspase-3. **e** Increased intraneuronal amyloid precursor protein (APP) as a sign of degeneration. IHC APP, Hippocampus. Inset: IHC APP control Hippocampus. (F) Accumulation of mitochondria in degeneration neurons. IHC VDAC1/Porin IHC, Hippocampus. Inset: IHC VDAC1/Porin IHC control Hippocampus. **g** Degenerating neuron with rounded swollen mitochondria. Cerebral cortex, EM. **h** The mitochondrial cristae are partially lost, consistent with cristolysis (arrows). Cerebral cortex, EM

At autopsy, no macroscopical changes were present in the central nervous system or other examined organs, and the histopathological changes were restricted to the brain. The main finding of this mitochondrial encephalopathy was severe acute neuronal degeneration and necrosis diffusely affecting the grey matter throughout the brain (Fig. 6a). Strongly eosinophilic, granular, degenerating neurons (Fig. 6b arrows) were present in both outer (III) layer and inner (V) pyramidal layers of the cerebral cortex as well as in the thalamic nuclei, pyramidal neurons of the Hippocampus and cerebellar Purkinje cells. Blood vessel wall cellularity was mildly increased (Fig. 6c) with occasional apoptotic cells (Fig. 6d) and perivascular astrocytic foot-processes were swollen, consistent with cytotoxic oedema on a subcellular level. Mild fibrillary astrogliosis was noted as an increased GFAP signal in the deep cortical cerebral layer. An increased punctuated intraneuronal accumulation of Aβ was evident in the affected brain when compared to control (Figs. 1c, 6e) and the affected neurons stained intensely for the mitochondrial outer membrane marker VDAC1/Porin (Fig. 6f). The degenerating neurons contained swollen mitochondria with mitochondrial cristolysis (Fig. 6g, h). There was no accumulation of ubiquitin-positive material in the tissue and the neuronal LC3 staining pattern was comparable to control sections.

### Defective mitochondrial respiratory function in the PITRM-affected tissues

**Fig. 7:**
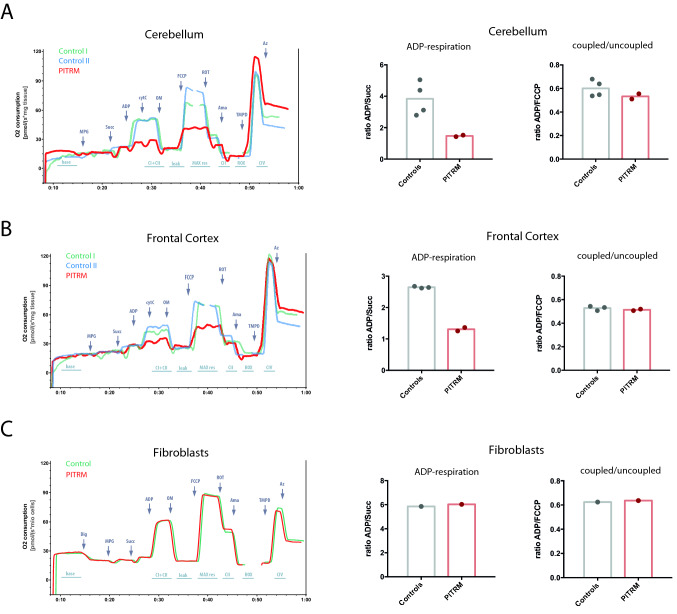
Analysis of mitochondrial function using high-resolution respirometry in the cerebellum (**a**), frontal cortex (**b**), and fibroblasts (**c**). Oxygen consumption traces and quantification in the cerebellum (**a**) and frontal cortex (**b**) of the affected dog compared to controls (*n* = 2). All tissue measurements performed in duplicates. **c** Oxygen consumption rate traces and quantification in immortalized fibroblasts from an affected dog and age-matched control

**Fig. 8:**
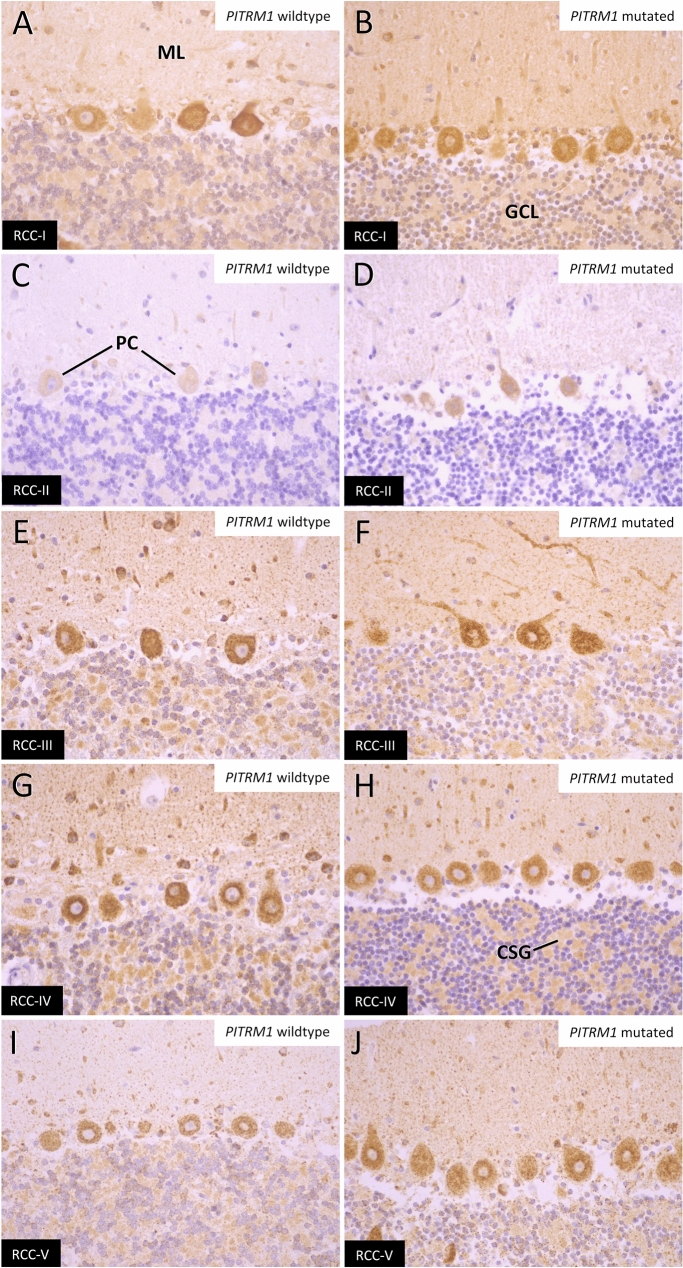
Respiratory chain complex proteins I–V in unaffected and affected dogs assessed by IHC. Despite mitochondrial dysfunction, expression of respiratory chain complexes I through V in indicator areas as cerebellar cortex is identical between PITRM1 wild-type (**a**, **c**, **e**, **g**, **i**) and mutant brains (**b**, **d**, **f**, **h**, **j**). *ML* molecular layer, *GCL* granular cell layer, *PC* Purkinje cells, *CSG* cerebellar synaptic glomeruli

To investigate mitochondrial respiratory chain function, we performed high-resolution respirometry in native brain tissue. We measured oxygen consumption in the cerebellum and frontal cortex of one affected (10 weeks of age) and two young control dogs: 9-month-old Siberian Husky euthanized due to epilepsy and 19-day-old Border Collie euthanized due to hydrocephalus. Both cerebellum and frontal cortex revealed a remarkable decrease in ADP-stimulated (complex I- and complex II-linked) respiration in the affected dog compared to controls (Fig. 7a, b). Comparison of this maximally coupled to uncoupled respiration (induced by FCCP) suggested that functional impairment was due to damaged mitochondria rather than only loss of mitochondrial respiratory complexes. Functional assessment of complex IV (CIV) showed comparable activity in both the affected dog and controls, suggesting functional impairment was not linked to a simple loss of the respiratory chain complexes but likely due to damaged mitochondria (Fig. 7a, b, left panel). Oxygen consumption rates in immortalized fibroblasts from the affected dog and one control were comparable, suggesting tissue-specific disease presentation (Fig. 7c). This hypothesis was supported by similar expression levels of mitochondrial respiratory chain complex proteins I–V in unaffected and affected dogs when evaluated by immunohistochemistry and western blotting (Fig. 8a–j, Online Resource 10).

### Modeling of the *PITRM* deletion in yeast

To evaluate whether the 6 bp deletion affects the PITRM1 activity, we modeled the variant in the yeast *Saccharomyces cerevisiae*, whose PITRM1 orthologue is *CYM1* (Alikhani et al. 2011b). Leu59 is conserved in yeast (Leu40), whereas Ser60 corresponds to an arginine (Arg41). We constructed two mutant alleles in the *CYM1* gene fused with the HA-tag: *cym1HA*^*R41S*^, in which Arg41 was changed with serine, the amino acid present in wild-type dogs, and *cym1*^*ΔL40R41*^, in which the two amino acids were deleted, as in the affected dogs. Wild-type and mutant alleles were introduced in the *cym1Δ* null strain.

**Fig. 9:**
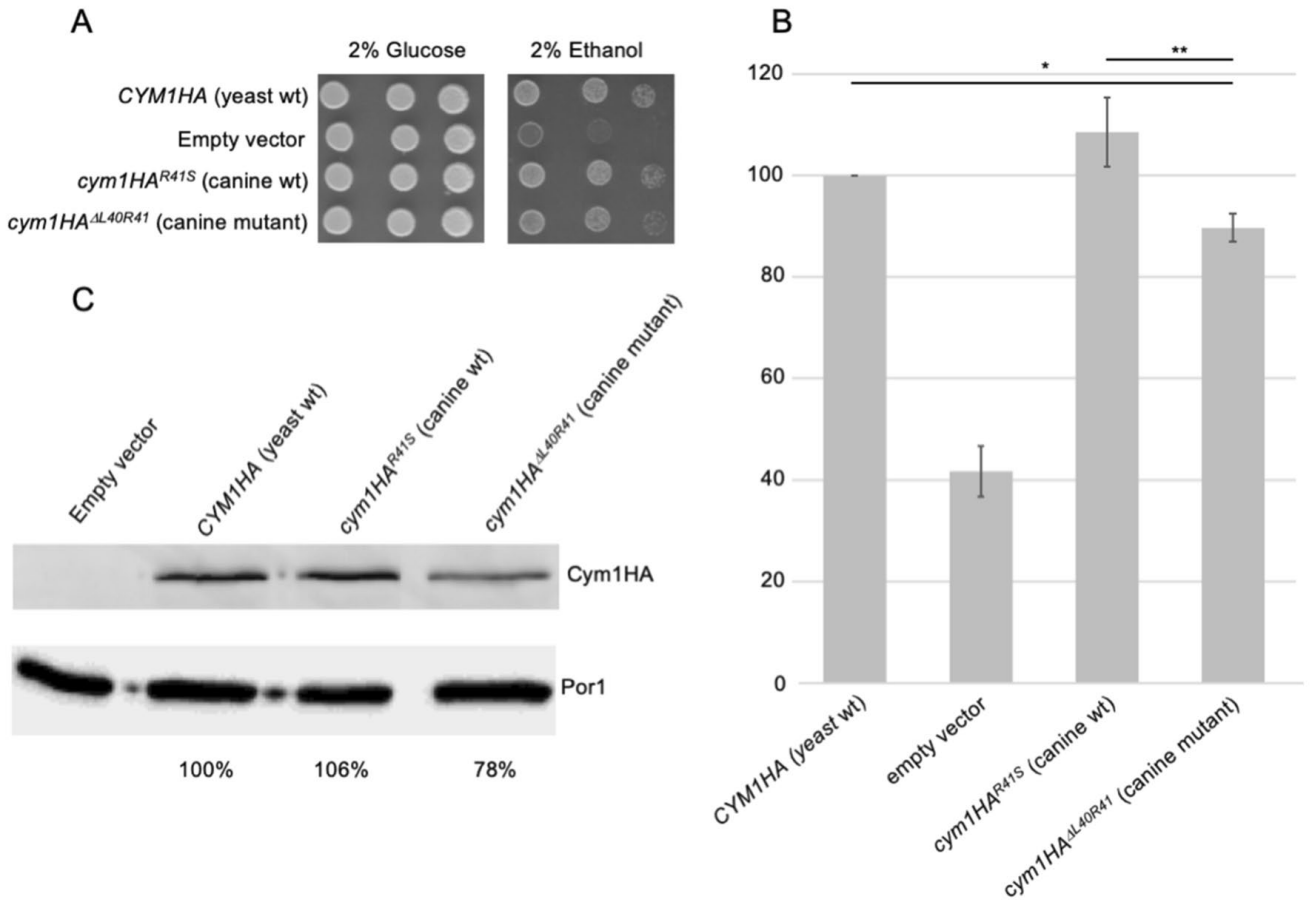
In vivo modeling of the mutation in yeast *Saccharomyces cerevisiae.* The strains harboring the wild-type *CYM1HA* allele (yeast wt), the *cym1HA*^*R41S*^ allele (canine wt), the *cym1*^*ΔL40R41*^ mutant allele (canine mutant) or the empty vector were analyzed. **a** Spot assay of wt and mutant strains on SC medium supplemented with glucose or ethanol: 1:3 dilutions were performed, starting from 1.5 × 10^4^ cells/spot. **b** Oxygen consumption rate (OCR) was measured on five independent clones for each strain and normalized for the cells dry weight. Data were then normalized to the OCR of the strain transformed with *CYM1HA* yeast wt allele and reported as mean ± SD. Statistical analysis was performed by one-way ANOVA followed by a post hoc Bonferroni test. *p < 0.05; **p < 0.01. **c** Western blot on total protein extracts. Cym1HA signal levels were measured, as reported in “Materials and methods”, and normalized for the Por1 signal and then to the *CYM1HA* yeast wt strain

Strains harboring *cym1HA*^*ΔL40R41*^ (canine mutant allele) showed a slight oxidative-growth defect on medium supplemented with non-fermentable carbon sources such as 2% ethanol (Fig. 9a) and a 15% decrease in the oxygen consumption rate compared to both the *CYM1HA* (yeast wt allele) and *cym1HA*^*R41S*^ (canine wt allele) strains (Fig. 9b). Western blot analysis of the *cym1HA*^*ΔL40R41*^ strain showed a decrease (approximately 20%) of the Cym1 mutant protein compared to the yeast wt and canine wt strains, suggesting impaired stability (Fig. 9c). Analysis in yeast, thus, confirm that the deletion has detrimental effects in an in vivo model.

### *PITRM1* deletion heterozygotes are clinically normal

*Pitrm*^−/−^ mice are embryonic lethal, while *Pitrm*^+^/^−^ mice survive but suffer from hind limb clasping, motor dysfunction and neurodegeneration with amyloid deposits (Brunetti et al. 2016). To find out whether the heterozygous dogs have any significant, slowly progressing neurological dysfunction, we performed a complete physical and neurological examination together with CBC and serum biochemistry on four heterozygous dogs at a mean age of 8.4 years. We did not observe any remarkable changes in any of the dogs.

## Discussion

We describe an early-onset lethal canine brain disorder characterized by severe progressive epilepsy with *status epilepticus*, mitochondrial dysfunction, accumulation of Aβ, and neurodegeneration due to a recessive deletion in the nuclear-encoded *PITRM1*. The affected puppies developed normally in the first 2–3 months of life before the onset of seizures. Although epileptic seizures have not been reported earlier in PITRM1 deficiency, the pathological findings in the affected dogs have overlapping features, such as intraneuronal amyloid precursor protein, with other species and induced organ models with PITRM1 deficiency (Brunetti et al. 2016; Langer et al. 2018; Perez et al. 2020). With a few weeks’ survival time, the affected dogs present clinically an intermediate model between the embryonic lethal mice and the slowly progressing childhood-onset human patients. Varying severity and progression of the disease could reflect species-specific differences in the mitochondrial PITRM function or relate to the nature of the described mutations with partial remaining catalytic activity.

The association between disease and PITRM1 variants in multiple species highlights the essential role of this protein for neuronal survival and maintenance of protein homeostasis. In dogs, the two-residue deletion in the N-terminal part of the protein resides in the region where the protein is predicted to transform from a short helix to a β-strand, suggesting that structural changes in the protein folding are likely. In turn, this could affect catalytic activity necessary for precursor processing and degradation. Immunohistochemistry suggested the PITRM protein levels not to be drastically altered, suggesting structural changes in the protein folding, potentially affecting the essential catalytic activity necessary for precursor processing and degradation. The PITRM1 transcript remains stable. When the deletion was modeled in yeast, a decrease in oxidative growth and in oxygen consumption rates were observed, demonstrating that this mutation affects the mitochondrial OXPHOS activity in vivo. The previously reported two human *PITRM1* variants, R183Q and T931M, resulted in the partial impairment of the activity or a nearly complete absence of the PITRM protein, respectively. Pitrm^−/−^-deficient mice were embryonic lethal while hemizygous Pitrm1^+^/^−^ mice survived and replicated many of the pathological features seen in human patients, including amyloidotic neurodegeneration (Brunetti et al. 2016). In contrast, we did not see any clinical signs in heterozygous dogs examined past the middle age at ~ 8 years of age.

The intraneuronal punctuate amyloid accumulation and neurological phenotype in humans, mice and dogs confirm the role of PITRM1 in the clearance of amyloid and the neurodegeneration that develops if the clearance fails. Interestingly, the clinical phenotype in dogs with a lethal *status epilepticus* occurring early in life is different from with the mild incoordination seen in hemizygous mice or the intellectual disability and ataxia in humans. Epilepsy, especially *status epilepticus*, is the primary determinant of clinical outcome and lesion progression in mitochondrial encephalopathies (Tzoulis and Bindoff 2012). Neurons with compromised energy production are prone to excitatory activity, and as epilepsy is an energy-demanding process, a vicious circle may evolve. Therefore, epilepsy, and its progression to *status epilepticus* in the dog, may negatively influence the canine phenotype.

The subcellular neuronal results of mitochondrial swelling and cristolysis, known as early necrosis events, are induced by anoxia and ATP loss. In association with ATP depletion, acidification of the cytoplasm occurs, correlating with increased cellular eosinophilia in HE sections. As there was no external cause for anoxia in the dog, the histological findings likely reflect the loss of oxidative phosphorylation in neurons at the cellular level. Cytotoxic oedema, as seen in astrocytes of the affected dog, has been linked to local energy loss and failure of keeping the electrochemical transmembrane gradient of sodium and potassium ions with a consequent influx of sodium and water (Tzoulis and Bindoff 2012). Energy deficiency may stimulate mitochondrial proliferation as increased numbers of mitochondria occur in mitochondrial angiopathy (Ohama et al. 1987) and in striated muscle cells in various mitochondrial myopathies. We found an increased IHC signal for mitochondrial membrane proteins in degenerating neurons but not in other examined tissues. Taken together, the neuropathological lesion pattern, cellular and subcellular findings show that the lesions are due to failure of oxidative metabolism and consequent cellular energy depletion, rather than ischemia from reduced perfusion or anoxia. The distribution of the neuronal necrosis in the dogs with PITRM deficiency is also quite different from the laminar cortical necrosis of ischemic encephalopathy and signals a systemic metabolic neuronal stress. Fibrillary astrogliosis is an unspecific, more chronic response to brain injury, and in this case, an older lesion than the acute neuronal necrosis. We did not detect other chronic neural lesions linked to PITRM1 dysfunction, such as accumulation of ubiquitinated material or increased autophagosomal amount. These results show a persistent subclinical disease preceding the critical neuronal damage that reflects the disease’s culmination as *status epilepticus*.

In summary, we describe a novel mitochondrial neurodegenerative disease with epileptic encephalopathy and demonstrate defective PITRM1 as the cause. This study expands the spectrum of diseases caused by PITRM1, and the affected canine breed will benefit from a genetic test to eradicate the condition from the population.

## Supplementary Information

Below is the link to the electronic supplementary material.

Online Resource 1. The number of genomes and exomes from different breeds used as control variant data set in filtering

Online Resource 2. A video showing of an affected male having an epileptic seizure at 7 weeks of age. Published with permission from Klára Kalatov under the terms of the Creative Commons Attribution 4.0 International License (http://creativecommons.org/licenses/by/4.0/)

Online Resource 3. A video showing an affected female having an epileptic seizure at 7 weeks of age. Published with permission from Klára Kalatov under the terms of the Creative Commons Attribution 4.0 International License (http://creativecommons.org/licenses/by/4.0/)

Online Resource 4. Regions of homozygosity obtained using homozygosity mapping of two affected and four unaffected dogs

Online Resource 5. The list of homozygous case-specific variants which remained after filtering the WES data of an affected Parson Russell Terrier

Online Resource 6. The list of homozygous case-specific variants with an effect on the protein which remained after filtering the WGS data of an affected Parson Russell Terrier

Online Resource 7. The case-specific structural variants and mobile element insertions

Online Resource 8. Results of the screening of the PITRM1 deletion in 28169 dogs from 374 breeds

Online Resource 9. A video of a 10-week-old affected puppy demonstrating focal-onset episodes

Online Resource 10. Representative western blots of PITRM1 in cortex and cerebellum from affected and unaffected dogs (A) Subunits of complex iv (mt-Co1), complex v (ATP5A) and complex ii (SDHA) did not show gross alterations. Mitochondrial mass marker TOM20 did show some variance in cerebellum of affected dog with not sufficient animals to allow any conclusions. Equal amounts of protein were loaded (stain-free total protein) with Vinculin as loading control. PITRM antibody was inconclusive on western blots

## Data Availability

Exome and whole-genome sequencing data from three dogs have been published in the SRA under the BioProject accession number PRJNA682160 https://www.ncbi.nlm.nih.gov/sra/?term=PRJNA682160.
