# Supplementary figures and images for: In-frame deletion in canine PITRM1 is associated with a severe early-onset epilepsy, mitochondrial dysfunction and neurodegeneration
Source: Hum Genet. 2021 Apr 9;140(11):1593–609. doi: 10.1007/s00439-021-02279-y (PMC8519929; doi:10.1007/s00439-021-02279-y)

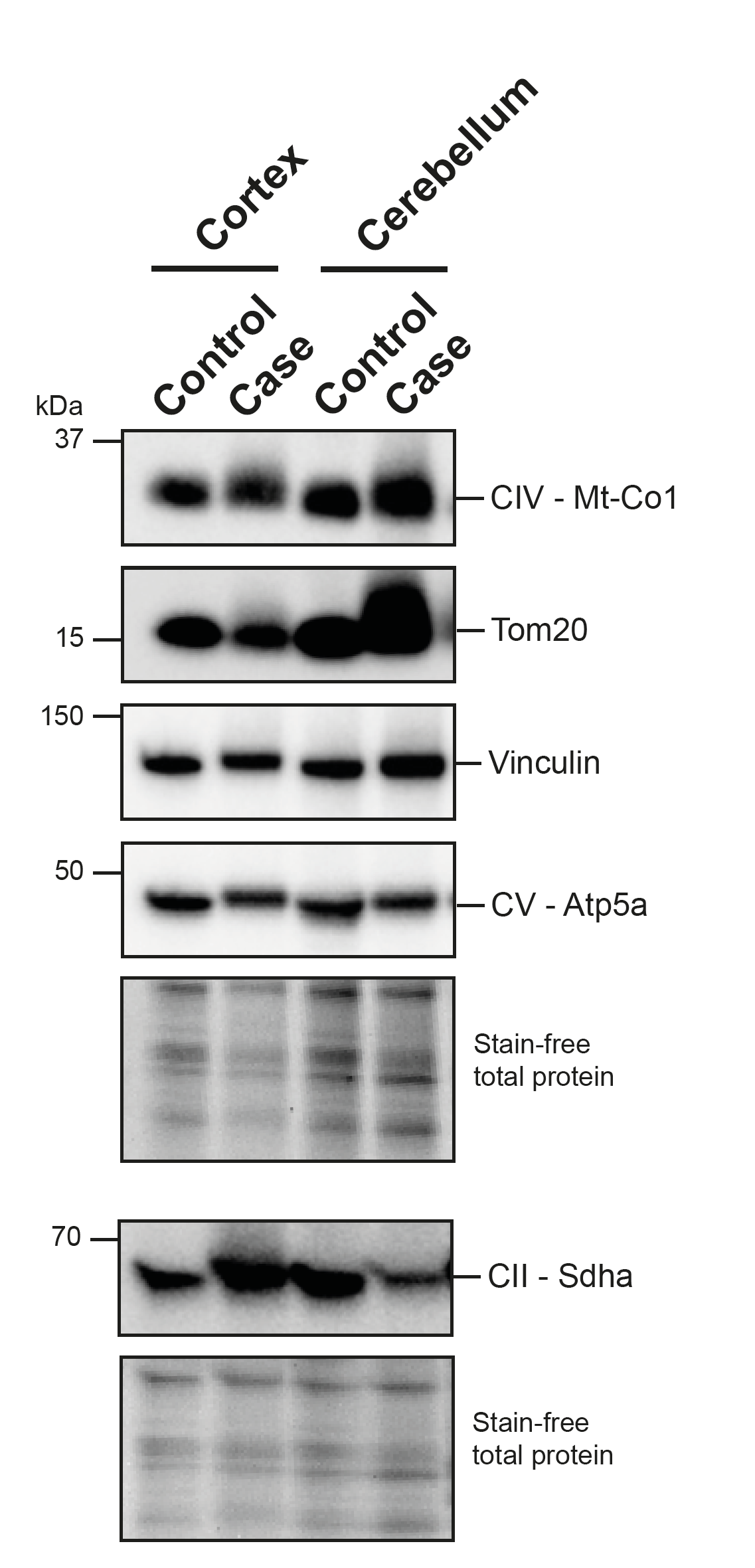

Supplement: Supplementary file 10 — Online Resource 10. Representative western blots of PITRM1 in cortex and cerebellum from affected and unaffected dogs (A) Subunits of complex iv (mt-Co1), complex v (ATP5A) and complex ii (SDHA) did not show gross alterations. Mitochondrial mass marker TOM20 did show some variance in cerebellum of affected dog with not sufficient animals to allow any conclusions. Equal amounts of protein were loaded (stain-free total protein) with Vinculin as loading control. PITRM antibody was inconclusive on western blots [file 439_2021_2279_MOESM10_ESM.tif]
